# *SPIND-TC*: an indexing method for two-color X-ray diffraction data

**DOI:** 10.1107/S2053273320001916

**Published:** 2020-04-02

**Authors:** Xuanxuan Li, Chufeng Li, Haiguang Liu

**Affiliations:** a Department of Engineering Physics, Tsinghua University, Beijing 100084, China; b Complex Systems Division, Beijing Computational Science Research Center, ZPark II, Haidian, Beijing 100193, China; c Department of Physics, Arizona State University, Tempe, AZ 85287, USA; d Center for Free-Electron Laser Science, Deutsches Elektronen-Synchrotron DESY, Notkestraße 85, 22607 Hamburg, Germany

**Keywords:** serial crystallography, two-color diffraction, indexing algorithm

## Abstract

Recent developments of two-color operation modes at X-ray free-electron laser facilities provide new research opportunities, such as X-ray pump/X-ray probe experiments and multiple-wavelength anomalous dispersion phasing methods. However, most existing indexing methods were developed for indexing diffraction data from monochromatic X-ray beams. Here, a new algorithm is presented for indexing two-color diffraction data, as an extension of the sparse-pattern indexing algorithm *SPIND*, which has been demonstrated to be capable of indexing diffraction patterns with as few as five peaks. The principle and implementation of the two-color indexing method, *SPIND-TC*, are reported in this paper. The algorithm was tested on both simulated and experimental data of protein crystals. The results show that the diffraction data can be accurately indexed in both cases. Source codes are publicly available at https://github.com/lixx11/SPIND-TC.

## Abstract 2

An auto-indexing method for two-color X-ray diffraction data is presented, which has been tested on both simulated and experimental protein diffraction data. The indexing yield is increased significantly compared with the previous approach using conventional indexers.

## 1. Introduction   

Over the past few years, serial femtosecond crystallography (SFX) has demonstrated the capabilities of determining three-dimensional macromolecular structures from microcrystals (Chapman *et al.*, 2011 ▸; Boutet *et al.*, 2012 ▸; Barends *et al.*, 2014 ▸; Kupitz *et al.*, 2014 ▸). Using femtosecond pulses of bright X-ray free-electron lasers (XFELs), diffraction signals are recorded from protein crystals at room temperature in the ‘diffraction-before-destruction’ approach (Solem, 1986 ▸; Neutze *et al.*, 2000 ▸). This scheme avoids the structure alteration in the cryogenic cooling process (Fraser *et al.*, 2011 ▸; Keedy *et al.*, 2014 ▸), which is frequently adopted to protect protein crystals from radiation damage in macromolecular diffraction experiments at synchrotron facilities.

In contrast to the conventional macromolecular crystallography using synchrotron light sources, where only one or a few large crystals are required for a complete data set using the oscillation approach, SFX experiments usually require thousands to millions of microcrystals to yield a complete data set. Since every crystal sample is destroyed after being illuminated by XFEL pulses, one crystal only produces a single still diffraction pattern. Each diffraction pattern corresponds to a slice of the three-dimensional reciprocal space. Due to the femtosecond duration and the narrow bandwidth of XFEL pulses, only partial intensities of Bragg spots are recorded on each diffraction pattern. Moreover, the variation of crystal size, shape and shot-to-shot XFEL intensity adds more fluctuation to diffraction signals. To reconstruct a reciprocal space with full intensities, each reflection needs to be sampled many times to average out the noise due to these stochastic factors, which in turn requires a large volume of diffraction data.

To reduce the sample consumption and experiment time, several attempts have been made to improve the throughput. At the Coherent X-ray Imaging (CXI) instrument (Liang *et al.*, 2015 ▸) of Linac Coherent Light Source (LCLS), researchers can refocus the transmitted beam that passes through the primary chamber, and conduct another independent experiment simultaneously (Boutet *et al.*, 2015 ▸). This serial operation doubles the data collection efficiency but can not reduce the sample consumption. The recent development of XFELs makes it possible to generate a pair of pulses with an adjustable separation of wavelength and time delay (Lutman *et al.*, 2013 ▸; Hara *et al.*, 2013 ▸). This two-color mode doubles the number of diffraction patterns collected from crystals, reducing both the beam time and the sample consumption. Gorel *et al.* applied the two-color approach in a multiple-wavelength anomalous dispersion experiment to determine the structure of lysozyme, and demonstrated that two-wavelength phases can be potentially more accurate than the single-wavelength case, since the second wavelength produces an additional independent measurement (Gorel *et al.*, 2017*b* ▸).

In SFX experiments, terabytes of diffraction data are collected and processed. Indexed patterns are merged to produce the intensity list for structure determination. At the first stage, the raw data are rapidly sorted and filtered by programs such as *Cheetah* (Barty *et al.*, 2014 ▸), *CASS* (Foucar, 2016 ▸) or *ClickX* (Li *et al.*, 2019*b* ▸). The resulting diffraction images can be further indexed and merged using the *CrystFEL* suite (White *et al.*, 2012 ▸). The program *indexamajig* of *CrystFEL* is integrated with several auto-indexers, such as *MOSFLM* (Powell, 1999 ▸), *DirAx* (Duisenberg, 1992 ▸) and *XDS* (Kabsch, 1988 ▸, 1993 ▸). Several new indexing algorithms have been developed recently. Brewster *et al.* developed a new indexing algorithm for sparse patterns, which showed good performance in indexing experimental patterns of peptide nanocrystals with small unit cells (Brewster *et al.*, 2015 ▸). Based on inter-spot vectors, *TakeTwo* (Ginn *et al.*, 2016 ▸) was shown to improve the indexing rate with the prior knowledge of unit-cell parameters for cubic, hexagonal and orthorhombic space groups. *SPIND* (Li *et al.*, 2019*a* ▸) is another prior-unit-cell-knowledge-based method, which searches the best rotation solutions using lengths and angles between pairs of Bragg spots and the origin point of the reciprocal space. *FELIX* (Beyerlein *et al.*, 2017 ▸) is able to index multiple crystals in serial crystallography patterns, and has been applied to simulated data sets of cubic, tetragonal and monoclinic crystals and experimental data sets from lysozyme microcrystals. The suite *cctbx.xfel* (Sauter *et al.*, 2013 ▸; Hattne *et al.*, 2014 ▸) represents an alternative set of SFX data processing programs, which can also index multiple crystals.

Here, we present an auto-indexing method for two-color diffraction patterns, *SPIND-TC*, as an extension of the sparse-data indexing method *SPIND*. This method has been tested on both simulated and experimental data sets, showing accurate indexing results. In particular, the indexing rate for an experimental data set is improved from 11.1% (as in the original work) to 50.9%.

## 2. Methods   

*SPIND-TC* is developed based on the sparse-pattern indexing algorithm *SPIND*, which finds the optimal orientation of a crystal using the prior knowledge of the unit cell as a reference. The core idea is summarized as follows:
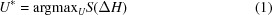


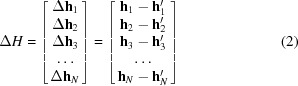


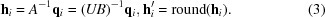



In equation (1), *S* is the scoring function to evaluate the matching quality between the observed peaks and predicted peaks by comparing the fractional Miller index, which is denoted by 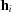, and the corresponding nearest integer Miller index 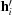 for the *i*th peak. The goal is to find a rotation matrix *U*, such that the scoring function *S* (the number of matched peaks) is maximized. *B* is the orthogonalization matrix of the reference lattice, and 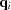 is the *i*th reciprocal vector. For any rotation matrix, Miller indices of all peaks are calculated. For the *i*th peak, if 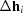, which is a 3-element tuple (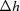, 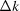, 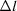), is small enough, it is considered as a matched peak. The matching criterion is formulated as below: 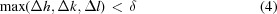
where δ is a user-specified parameter, and is set to 0.25 by default. In other words, if the largest deviation of Miller index is within 0.25, the observed peak is considered to match the predicted peak determined by *U* and *B*. The solution with most matched peaks, *i.e.* largest *S*, is considered as the best solution.

If the raw diffraction patterns (or the Miller indices derived from each diffraction pattern) were directly used to compare with the reference patterns, then many reference patterns are required to sample all possible orientations. This approach is impractical due to the high demands in computation. The *SPIND* algorithm first converts the Bragg vectors to the representation that is independent of orientations, using [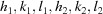, 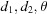] to generate a reference table [see the *SPIND* paper (Li *et al.*, 2019*a* ▸) and Appendix *A* [App appa] for details].

**Figure 1:**
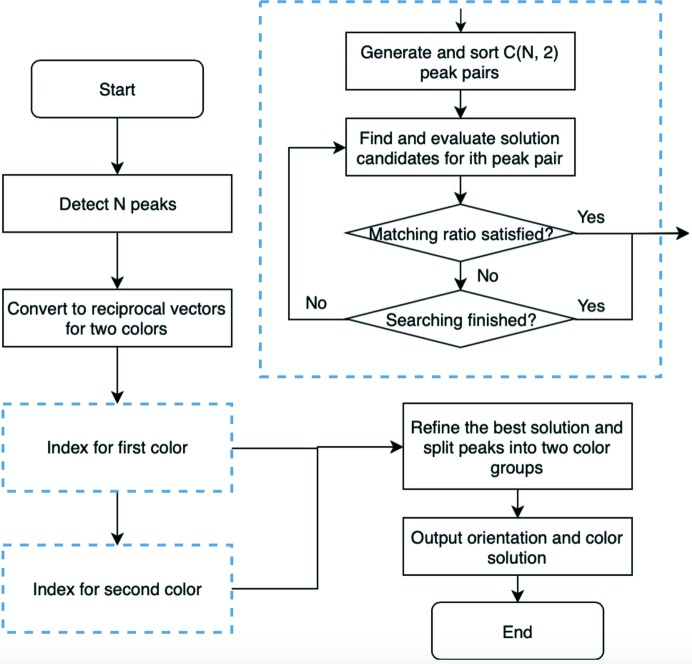
Workflow of SPIND-TC. The indexing is performed for two colors independently, which is shown in two blue dashed boxes. The best rotation matrix is refined to obtain the final indexing solution and used to split the peaks into two color groups.

For two-color pattern indexing, the scoring function is modified to take the two different wavelengths into account. A peak matched to either color is regarded as a matched peak. The workflow is described in Fig. 1 ▸.

First, the peaks are detected on the two-dimensional diffraction images. The reciprocal vectors are calculated with given geometry parameters. The indexing (or searching) is conducted on the corresponding reciprocal vectors for two wavelengths independently.

The searching is a reference-matching process (dashed boxes in Fig. 1 ▸). The reference in *SPIND*/*SPIND-TC* is a pre-calculated table for the given space group and unit-cell parameters in the specified resolution range. Each Miller index pair is represented using three parameters, two vector lengths and the angle between the two vectors, hereafter denoted as a reference triple. The reference table is used to match peak pairs from diffraction patterns. The vector lengths and angles are used to narrow down the potential Bragg vectors to a small set, and then the rotation matrix is calculated using the other information in each reference to identify the orientation and assess the matching quality using the Miller indices.

For a pattern with *N* peaks, 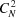 peak pairs can be generated and sorted by intensity, resolution or signal-to-noise ratio (SNR). Users can select top *k* peak pairs for matching. The two lengths and one angle for each pair, denoted as an observed triple, are used to match the entries in the reference table within the given tolerance. A rotation matrix *U* can be calculated for each matched entry. Each rotation solution is evaluated by the scoring function. In monochromatic cases, a peak with small 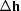 is considered as a matched peak. In two-color cases, a peak with either small 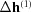 or small 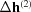 (1, 2 are used to denote the two colors) is considered as a matched peak.

After the reference-based indexing, the peak list is divided into two groups, 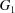 and 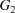, according to the color probabilities. The probability of the *i*th peak resulting from color *j* is assigned as below: 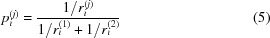


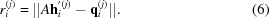
Finally a global refinement is performed to optimize the final solution for the following objective function using *scipy.optimize* (Jones *et al.*, 2001–2020 ▸):
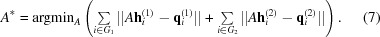



## 3. Results   

### 3.1. Indexing simulated two-color diffraction data   

**Figure 2:**
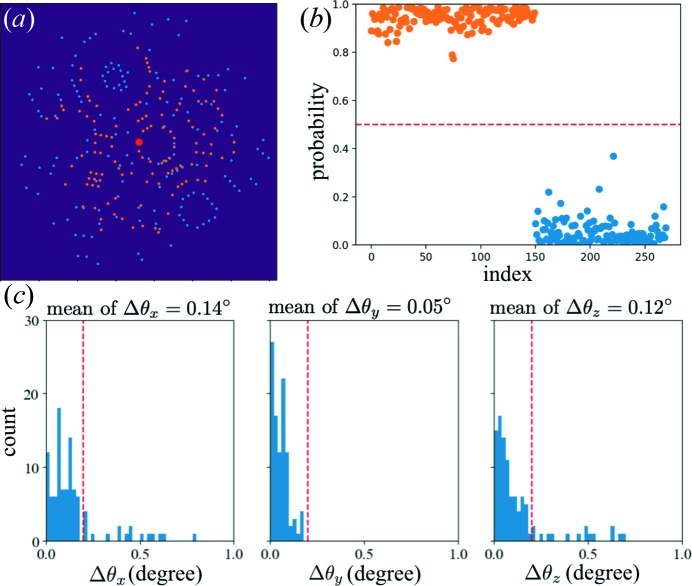
Indexing results on the simulated data set. (*a*) A typical simulated two-color diffraction pattern using 7 keV (orange peaks) and 9 keV (blue peaks) XFELs in the orientation specified by Euler angles 10, 20, 30°. (*b*) Probabilities of 7 keV for all peaks in the simulated pattern (*a*). The probabilities larger than 0.5 corresponding to the peaks of 7 keV (orange). (*c*) Distribution of orientation errors for each Euler angle. Most of the angle errors are smaller than 0.2°.

To validate the ability of *SPIND-TC* to index two-color diffraction patterns, a simulated data set was generated from protein crystals [Protein Data Bank (PDB) code 5m2t, Prokofev *et al.*, unpublished] at random orientations. The protein crystal has a *P*1 space group and a unit cell of *a* = 64.3, *b* = 72.0, *c* = 89.2 Å and α = 110.6, β = 107.5, γ = 85.8°. The diffraction patterns are simulated on a 1440 × 1440-pixel virtual detector with a pixel size of 100 × 100 µm. The detector is placed 0.1 m away from the sample downstream of the incident beam and perpendicular to the beam. Because the absolute intensity values are not used for indexing simulated data, a simplified model is used to calculate the diffraction patterns, where structure factors, excitation error and signal noise are not included. The lattice points are modeled as spheres with fixed radius. A lattice point is considered as an excited spot if it is intercepted by the Ewald spheres of photon energy at 7 or 9 keV, and the corresponding peak coordinate and the photon energy are registered. Each simulated diffraction pattern consists of multiple peaks from two photon energies on the two-dimensional detector [see Fig. 2 ▸(*a*) for an example]. All 100 simulated patterns were indexed successfully with correct orientation solutions (Fig. 2 ▸).

### 3.2. Indexing experimental two-color diffraction data   

To further investigate the performance of *SPIND-TC* on actual experimental data, we carried out an indexing test on data set ID 66 (Gorel *et al.*, 2017*a* ▸) in the Coherent X-ray Imaging Data Bank (CXIDB) (Maia, 2012 ▸). This data set was collected at SACLA (SPring-8 Angstrom Compact free-electron LAser) in 2016, and contains 208 373 diffraction patterns identified with *Cheetah*. In this experiment, 7 and 9 keV XFEL pulses were used to produce the diffraction images. Since no available program had been developed to index such two-color diffraction data by the time of the work, Gorel *et al.* used a two-round indexing approach, which utilizes the fact that the two-color images usually consist of two sets of diffraction peaks resulting from XFEL pulses with different fluences, so that the observed Bragg spots can be grouped by intensity. They first used a high-intensity threshold to detect strong peaks and tried indexing assuming 7 or 9 keV photon energy independently. After the first round of indexing, the diffraction images were reprocessed to extract peaks using a low threshold, resulting in more peaks including the ones with weaker signals. Peaks that can be indexed in the first round are masked out for the second round of indexing. The remaining peaks are indexed assuming the other photon energy that was not used for the first round. Using this detect–index–detect–index approach, which is referred to as the *CrystFEL-TC* approach in this article, 23 144 patterns were indexed for both colors.

However, the peak intensities are not only affected by the intensity of the XFEL pulses, but also by characteristics of crystal samples, including structure factors, partiality and mosaicity. The previous approach relies on the intensity-based peak grouping, resulting in low data efficiency. The *SPIND-TC* algorithm overcomes the dependency on correctly sorting the Bragg peaks based on intensity information. It searches the optimal orientation solution for two colors based on the location of peaks in a single round of data processing, and could improve the indexing rate significantly.

**Figure 3:**
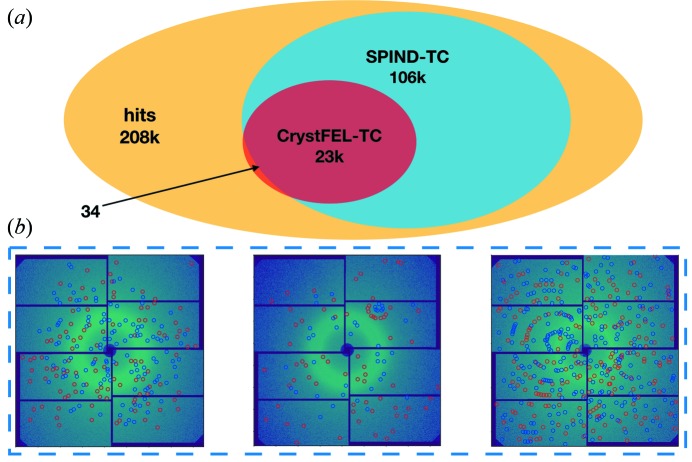
(*a*) Indexing results with *CrystFEL-TC* and *SPIND-TC* on the two-color data set. In 208 000 hits, *CrystFEL-TC* indexed 23 000 two-color patterns, while *SPIND-TC* can index 106 000 patterns, covering almost all of the patterns indexed by *CrystFEL-TC* (except 34 patterns). (*b*) Representative patterns that can only be indexed with *SPIND-TC*.

To be consistent with the workflow of Gorel *et al.*, *indexamajig* was used to detect peaks with an intensity threshold of 150 and SNR of 3 to include both strong and weak Bragg peaks. The peak lists were then processed by *SPIND-TC*. The peaks that were successfully classified into two colors were saved to hdf5 files with the associated indexing solution. Finally, we used *indexamajig* to check all indexing solutions on the classified peaks, and wrote results to stream files that are compatible with *CrystFEL*. By following this workflow, 106 154 images were indexed successfully, about 3.6 times more than that using the previous approach (Fig. 3 ▸).

**Figure 4:**
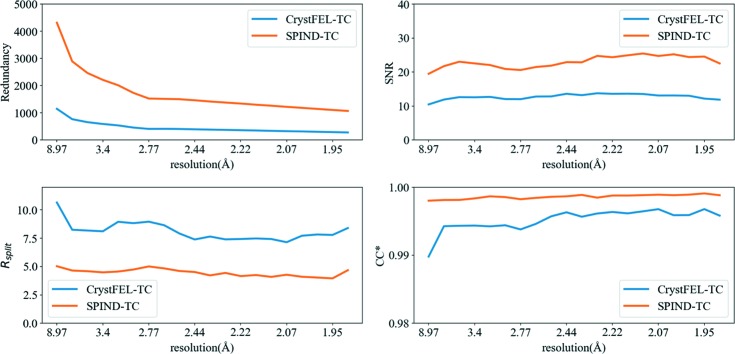
Binned figures-of-merit (FOMs) comparison between *SPIND-TC* and *CrystFEL-TC*. *SPIND-TC* performs better than the *CrystFEL-TC* method in all FOMs, including redundancy, SNR, *R*_split_ and CC*.

To compare the merged data quality between *SPIND-TC* and *CrystFEL-TC*, we followed the instruction in the original paper to index the 9 keV data set with *MOSFLM* and *DirAx*, and obtained 30 663 indexed patterns. The indexing results of *SPIND-TC* and *CrystFEL-TC* were merged using *partialator*. Since *SPIND-TC* had more patterns indexed, the redundancy was improved significantly, as well as *R*_split_ and CC*. A higher SNR indicates that *SPIND-TC* indexed more accurately than the *CrystFEL-TC* approach (Fig. 4 ▸).

### 3.3. Speed test   

**Figure 5:**
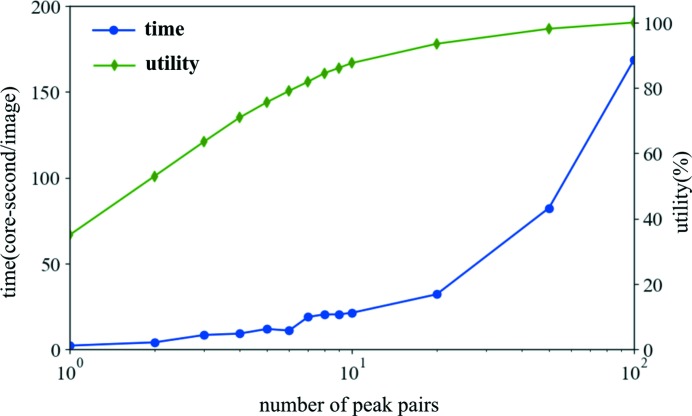
Performance of *SPIND-TC* at various matching pairs. The blue line shows the processing speed in core-seconds, while the green line represents the utility. If 100 peak pairs are searched in *SPIND-TC*, 170 core-seconds is required to index a single image, resulting in 6192 images indexed.

*SPIND-TC* is implemented in Python, but the throughput of indexing on experimental protein data is reasonably high. A series of tests were conducted to evaluate the processing speed of *SPIND-TC*. We used 10, 000 two-color images from the CXIDB 66 repository for all the speed tests. A reference table containing reflections below 5 Å was generated for indexing. The matching tolerances for vector lengths and angles were set to 0.0025 Å and 1°. All peaks were sorted by SNR values. The numbers of peak pairs selected for finding rotation solutions were tested in the range from one to 100, and the corresponding indexing time was 2.2 to 168.7 core-seconds per pattern as shown in Fig. 5 ▸. The utility is defined as the percentage of indexed patterns out of all indexable patterns. In this case, the number of all indexable patterns was determined to be 6192. Users can select a proper number of peak pairs according to the available computation power. With limited computational facility, it is recommended to start with a small number of peak pairs for matching, *e.g.* five, as the matching targets. It is found that a high utility can still be achieved with fast processing speed by using only five peak pairs from each pattern.

## 4. Discussion and conclusions   

Two-color modes of XFEL provide new opportunities and also bring challenges for serial crystallography. In two-color experiments, the data collection rate is doubled since one image contains two diffraction patterns. For small energy separation, such as  1%, which is feasible at LCLS, it is anticipated that such two-color data can be indexed by monochromatic methods as well as *SPIND-TC*. This was confirmed by a simulation test with 9 and 9.1 keV energy, where all images could be successfully indexed by *MOSFLM* and *SPIND-TC*. The problem for such data is the peak integration for the overlapped spots in the low-resolution region, which requires accurate orientation refinement and careful intensity deconvolution. On the other hand, such data can be analyzed using the pink-beam diffraction method, which uitlizes broader energy bandwidth (up to 5% 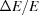) (Meents *et al.*, 2017 ▸).

In this work, we focused on the large energy separation cases (*e.g.* 7 and 9 keV). The two Ewald spheres sample different regions of the reciprocal space and thus are difficult to index using indexing algorithms which are developed for indexing diffraction patterns from monochromatic X-rays. The indexing method *SPIND-TC* presented here perfectly fulfills the requirements for indexing two-color diffraction patterns. It does not depend on intensity sorting of Bragg spots, and has been tested on both simulated and experimental protein data. The indexing rate for experimental data was increased by approximately 3.6 times compared with the previously reported results. Source codes are publicly available at https://github.com/lixx11/SPIND-TC.

## Appendix A Implementation of rotation solution searching in *SPIND/SPIND-TC*

For *N* reciprocal-lattice points within a specified resolution range,  (the number of possible combinations of the vector pairs) entries are pre-calculated and stored in the reference table [for one crystal sample with known unit-cell parameters, only one reference table is generated, instead of discretely sampling the whole three-dimensional reciprocal lattice with sufficiently small angular intervals, the strategy used in many reference matching algorithms such as the expand–maximize–compress (EMC) method (Ayyer *et al.*, 2015 ▸)]. Each entry in the reference table consists of two *hkl* indices, two lengths and one angle of a reciprocal-lattice pair, which are shown as follows:

The rotation solution searching is a matching process. Using the experimental geometry and XFEL wavelength information, two lengths and one angle are calculated for each experimental Bragg spot pair in the reciprocal space. These experimental triples are then used to match the entries in the reference table within the given tolerance:

For each match between the experimental entry (two vector lengths and one angle) and the reference entry, a rotation matrix *U* was computed based on the vectors ( ). The rotation matrix *U* represents the appropriate orientation relation between the experimental lattice and the reference lattice in reciprocal space:

The rotation matrix *U* is calculated in two steps. In the first step, , which represents the mapping from the plane of  and  to the plane of  and , is calculated using the axis–angle method:

where  and  are normal vectors for the  plane and the  plane.  can be calculated with the axis  [] and the angle .

After the first step, we can calculate  and , where  and  are in the same plane of  and .

In the second step, we can calculate , which transforms  and  to  and :

This is also done with the axis–angle method, where the axis is  and the angle is the average value of  and .

Finally, the rotation matrix *U* can be calculated from the product of  and ,

## References

[bb1] Ayyer, K., Philipp, H. T., Tate, M. W., Wierman, J. L., Elser, V. & Gruner, S. M. (2015). *IUCrJ*, **2**, 29–34. 10.1107/S2052252514022313 PMC4285878 25610625

[bb2] Barends, T. R. M., Foucar, L., Botha, S., Doak, R. B., Shoeman, R. L., Nass, K., Koglin, J. E., Williams, G. J., Boutet, S., Messerschmidt, M. & Schlichting, I. (2014). *Nature*, **505**, 244–247. 10.1038/nature12773 24270807

[bb3] Barty, A., Kirian, R. A., Maia, F. R. N. C., Hantke, M., Yoon, C. H., White, T. A. & Chapman, H. (2014). *J. Appl. Cryst.* **47**, 1118–1131. 10.1107/S1600576714007626 PMC4038800 24904246

[bb4] Beyerlein, K. R., White, T. A., Yefanov, O., Gati, C., Kazantsev, I. G., Nielsen, N. F.-G., Larsen, P. M., Chapman, H. N. & Schmidt, S. (2017). *J. Appl. Cryst.* **50**, 1075–1083. 10.1107/S1600576717007506 PMC5541352 28808433

[bb5] Boutet, S., Foucar, L., Barends, T. R. M., Botha, S., Doak, R. B., Koglin, J. E., Messerschmidt, M., Nass, K., Schlichting, I., Seibert, M. M., Shoeman, R. L. & Williams, G. J. (2015). *J. Synchrotron Rad.* **22**, 634–643. 10.1107/S1600577515004002 PMC4416680 25931079

[bb6] Boutet, S., Lomb, L., Williams, G. J., Barends, T. R. M., Aquila, A., Doak, R. B., Weierstall, U., DePonte, D. P., Steinbrener, J., Shoeman, R. L., Messerschmidt, M., Barty, A., White, T. A., Kassemeyer, S., Kirian, R. A., Seibert, M. M., Montanez, P. A., Kenney, C., Herbst, R., Hart, P., Pines, J., Haller, G., Gruner, S. M., Philipp, H. T., Tate, M. W., Hromalik, M., Koerner, L. J., van Bakel, N., Morse, J., Ghonsalves, W., Arnlund, D., Bogan, M. J., Caleman, C., Fromme, R., Hampton, C. Y., Hunter, M. S., Johansson, L. C., Katona, G., Kupitz, C., Liang, M., Martin, A. V., Nass, K., Redecke, L., Stellato, F., Timneanu, N., Wang, D., Zatsepin, N. A., Schafer, D., Defever, J., Neutze, R., Fromme, P., Spence, J. C. H., Chapman, H. N. & Schlichting, I. (2012). *Science*, **337**, 362–364.

[bb7] Brewster, A. S., Sawaya, M. R., Rodriguez, J., Hattne, J., Echols, N., McFarlane, H. T., Cascio, D., Adams, P. D., Eisenberg, D. S. & Sauter, N. K. (2015). *Acta Cryst.* D**71**, 357–366. 10.1107/S1399004714026145 PMC4321489 25664747

[bb8] Chapman, H. N., Fromme, P., Barty, A., White, T. A., Kirian, R. A., Aquila, A., Hunter, M. S., Schulz, J., DePonte, D. P., Weierstall, U., Doak, R. B., Maia, F. R. N. C., Martin, A. V., Schlichting, I., Lomb, L., Coppola, N., Shoeman, R. L., Epp, S. W., Hartmann, R., Rolles, D., Rudenko, A., Foucar, L., Kimmel, N., Weidenspointner, G., Holl, P., Liang, M., Barthelmess, M., Caleman, C., Boutet, S., Bogan, M. J., Krzywinski, J., Bostedt, C., Bajt, S., Gumprecht, L., Rudek, B., Erk, B., Schmidt, C., Hömke, A., Reich, C., Pietschner, D., Strüder, L., Hauser, G., Gorke, H., Ullrich, J., Herrmann, S., Schaller, G., Schopper, F., Soltau, H., Kühnel, K.-U., Messer­schmidt, M., Bozek, J. D., Hau-Riege, S. P., Frank, M., Hampton, C. Y., Sierra, R. G., Starodub, D., Williams, G. J., Hajdu, J., Timneanu, N., Seibert, M. M., Andreasson, J., Rocker, A., Jönsson, O., Svenda, M., Stern, S., Nass, K., Andritschke, R., Schröter, C.-D., Krasniqi, F., Bott, M., Schmidt, K. E., Wang, X., Grotjohann, I., Holton, J. M., Barends, T. R. M., Neutze, R., Marchesini, S., Fromme, R., Schorb, S., Rupp, D., Adolph, M., Gorkhover, T., Andersson, I., Hirsemann, H., Potdevin, G., Graafsma, H., Nilsson, B. & Spence, J. C. H. (2011). *Nature*, **470**, 73–77.

[bb9] Duisenberg, A. J. M. (1992). *J. Appl. Cryst.* **25**, 92–96.

[bb10] Foucar, L. (2016). *J. Appl. Cryst.* **49**, 1336–1346. 10.1107/S1600576716009201 PMC4970498 27504079

[bb11] Fraser, J. S., van den Bedem, H., Samelson, A. J., Lang, P. T., Holton, J. M., Echols, N. & Alber, T. (2011). *Proc. Natl Acad. Sci. USA*, **108**, 16247–16252. 10.1073/pnas.1111325108 PMC3182744 21918110

[bb12] Ginn, H. M., Roedig, P., Kuo, A., Evans, G., Sauter, N. K., Ernst, O. P., Meents, A., Mueller-Werkmeister, H., Miller, R. J. D. & Stuart, D. I. (2016). *Acta Cryst.* D**72**, 956–965. 10.1107/S2059798316010706 PMC4973211 27487826

[bb13] Gorel, A., Motomura, K., Fukuzawa, H., Doak, R. B., Grünbein, M. L., Hilpert, M., Inoue, I., Kloos, M., Kovács, G. N., Nango, E., Nass, K., Roome, C. M., Shoeman, R. L., Tanaka, R., Tono, K., Foucar, L., Joti, Y., Yabashi, M., Iwata, S., Ueda, K., Barends, T. R. M. & Schlichting, I. (2017*a*). *Scientific Data*, **4**, 170188. 10.1038/sdata.2017.188 PMC5726314 29231920

[bb14] Gorel, A., Motomura, K., Fukuzawa, H., Doak, R. B., Grünbein, M. L., Hilpert, M., Inoue, I., Kloos, M., Kovácsová, G., Nango, E., Nass, K., Roome, C. M., Shoeman, R. L., Tanaka, R., Tono, K., Joti, Y., Yabashi, M., Iwata, S., Foucar, L., Ueda, K., Barends, T. R. M. & Schlichting, I. (2017*b*). *Nat. Commun.* **8**, 1170. 10.1038/s41467-017-00754-7 PMC5660077 29079797

[bb15] Hara, T., Inubushi, Y., Katayama, T., Sato, T., Tanaka, H., Tanaka, T., Togashi, T., Togawa, K., Tono, K., Yabashi, M. & Ishikawa, T. (2013). *Nat. Commun.* **4**, 2919. 10.1038/ncomms3919 24301682

[bb16] Hattne, J., Echols, N., Tran, R., Kern, J., Gildea, R. J., Brewster, A. S., Alonso-Mori, R., Glöckner, C., Hellmich, J., Laksmono, H., Sierra, R. G., Lassalle-Kaiser, B., Lampe, A., Han, G., Gul, S., DiFiore, D., Milathianaki, D., Fry, A. R., Miahnahri, A., White, W. E., Schafer, D. W., Seibert, M. M., Koglin, J. E., Sokaras, D., Weng, T.-C., Sellberg, J., Latimer, M. J., Glatzel, P., Zwart, P. H., Grosse-Kunstleve, R. W., Bogan, M. J., Messerschmidt, M., Williams, G. J., Boutet, S., Messinger, J., Zouni, A., Yano, J., Bergmann, U., Yachandra, V. K., Adams, P. D. & Sauter, N. K. (2014). *Nat. Methods*, **11**, 545–548.

[bb17] Jones, E., Oliphant, T. & Peterson, P. (2001–2020). *SciPy: open source scientific tools for Python.* http://www.scipy.org/.

[bb18] Kabsch, W. (1988). *J. Appl. Cryst.* **21**, 67–72.

[bb19] Kabsch, W. (1993). *J. Appl. Cryst.* **26**, 795–800.

[bb20] Keedy, D. A., van den Bedem, H., Sivak, D. A., Petsko, G. A., Ringe, D., Wilson, M. A. & Fraser, J. S. (2014). *Structure*, **22**, 899–910. 10.1016/j.str.2014.04.016 PMC4082491 24882744

[bb21] Kupitz, C., Basu, S., Grotjohann, I., Fromme, R., Zatsepin, N. A., Rendek, K. N., Hunter, M. S., Shoeman, R. L., White, T. A., Wang, D., James, D., Yang, J.-H., Cobb, D. E., Reeder, B., Sierra, R. G., Liu, H., Barty, A., Aquila, A., Deponte, D., Kirian, R. A., Bari, S., Bergkamp, J. J., Beyerlein, K. R., Bogan, M. J., Caleman, C., Chao, T.-C., Conrad, C. E., Davis, K. M., Fleckenstein, H., Galli, L., Hau-Riege, S. P., Kassemeyer, S., Laksmono, H., Liang, M., Lomb, L., Marchesini, S., Martin, A. V., Messerschmidt, M., Milathianaki, D., Nass, K., Ros, A., Roy-Chowdhury, S., Schmidt, K., Seibert, M. M., Steinbrener, J., Stellato, F., Yan, L., Yoon, C., Moore, T. A., Moore, A. L., Pushkar, Y., Williams, G. J., Boutet, S., Doak, R. B., Weierstall, U., Frank, M., Chapman, H. N., Spence, J. C. H. & Fromme, P. (2014). *Nature*, **513**, 261–265.

[bb22] Li, C., Li, X., Kirian, R., Spence, J. C. H., Liu, H. & Zatsepin, N. A. (2019*a*). *IUCrJ*, **6**, 72–84. 10.1107/S2052252518014951 PMC6327178 30713705

[bb23] Li, X., Li, C. & Liu, H. (2019*b*). *J. Appl. Cryst.* **52**, 674–682. 10.1107/S1600576719005363 PMC6557179 31236097

[bb24] Liang, M., Williams, G. J., Messerschmidt, M., Seibert, M. M., Montanez, P. A., Hayes, M., Milathianaki, D., Aquila, A., Hunter, M. S., Koglin, J. E., Schafer, D. W., Guillet, S., Busse, A., Bergan, R., Olson, W., Fox, K., Stewart, N., Curtis, R., Miahnahri, A. A. & Boutet, S. (2015). *J. Synchrotron Rad.* **22**, 514–519. 10.1107/S160057751500449X PMC4416669 25931062

[bb25] Lutman, A., Coffee, R., Ding, Y., Huang, Z., Krzywinski, J., Maxwell, T., Messerschmidt, M. & Nuhn, H.-D. (2013). *Phys. Rev. Lett.* **110**, 134801. 10.1103/PhysRevLett.110.134801 23581326

[bb26] Maia, F. R. N. C. (2012). *Nat. Methods*, **9**, 854–855. 10.1038/nmeth.2110 22936162

[bb27] Meents, A., Wiedorn, M., Srajer, V., Henning, R., Sarrou, I., Bergtholdt, J., Barthelmess, M., Reinke, P., Dierksmeyer, D., Tolstikova, A., Schaible, S., Messerschmidt, M., Ogata, C. M., Kissick, D. J., Taft, M. H., Manstein, D. J., Lieske, J., Oberthuer, D., Fischetti, R. F. & Chapman, H. N. (2017). *Nat. Commun.* **8**, 1281. 10.1038/s41467-017-01417-3 PMC5668288 29097720

[bb28] Neutze, R., Wouts, R., van der Spoel, D., Weckert, E. & Hajdu, J. (2000). *Nature*, **406**, 752–757. 10.1038/35021099 10963603

[bb29] Powell, H. R. (1999). *Acta Cryst.* D**55**, 1690–1695. 10.1107/s0907444999009506 10531518

[bb30] Sauter, N. K., Hattne, J., Grosse-Kunstleve, R. W. & Echols, N. (2013). *Acta Cryst.* D**69**, 1274–1282. 10.1107/S0907444913000863 PMC3689530 23793153

[bb31] Solem, J. C. (1986). *J. Opt. Soc. Am. B*, **3**, 1551–1565.

[bb32] White, T. A., Kirian, R. A., Martin, A. V., Aquila, A., Nass, K., Barty, A. & Chapman, H. N. (2012). *J. Appl. Cryst.* **45**, 335–341.

